# Cases of Cynanche Trachealis

**Published:** 1830-01-01

**Authors:** 


### XXXIV.

### CASES OF CYNANCHE TRACHEALIS.

Dr. Jackson, Assistant Professor of Medicine and Clinical Practice in the University of Pennsylvania, has published three very interesting cases of cynanche trachealis in the American Journal of the Medical Sciences for August last. The deductions which he draws from them appear to us to be so just and of so practical a nature, that we cannot refrain from making our readers acquainted with the substance of the paper.

*Case* 1. "On the 7th of February, 1829, I was requested to visit, in consultation, E.M. a female child four years of age. She had enjoyed good health, was well nourished, and possessed full embonpoint.

"A week previous this child had been attacked, I was informed by Dr. M. who had attended it, with catarrh and inflammation of the tonsils. Emetics and purgatives had been administered; they had operated favourably; and an apparent amendment had ensued. On the 5th the respiration became stridulous and laboured—venesection ad deliquium was practised with the warm bath; which again produced an abatement of the symptoms. On the morning of the 7th, when I first saw the case, the respiration had again become exceedingly laboured, accompanied with the peculiar sound of croup, stridulous, dry, or unattended with any mucous rattle; cough dry. Examination of the throat showed the tonsils enlarged, a small ulcer on each, the posterior fauces red, but no appearance of membranous exudation, as is often seen in cases of croup commencing in cynanche tonsillaris.

"The treatment directed was sixty leeches to the throat, and warm bath—with three grains of colomel every half hour—after the leeches, a blister to the throat. No improvement was manifested. In the evening twenty drops of hive syrup every hour added to the former treatment, and calomel reduced to one grain every hour. At the same time muriatic acid was applied to the fauces by means of a brush, as recommended by Bretonneau, in the belief, that, although no exudation was discernible in the fauces, it existed in the larynx, and blocked up the glottis, into which the air was introduced with great difficulty."

Next day there was some improvement, the mucous secretion being established in the fauces and trachea, and the respiration less croupy. Mucous rattle in both lungs—thorax resonant—pulse frequent—cheeks flushed. A blister was applied to each side of the chest, the calomel continued, and at night a pediluvium with poultices to the legs. The little patient became worse during the night, and on exploring the chest it was found that although still resonant on percussion, the respiratory murmur had disappeared. Towards noon of the 9th the child expired suffocated.

*Dissection by Dr. Horner, 24 hours after death.* "Present Dr. Jackson; weather cold, and no visible putrefaction. On opening the thorax, the lungs did not collapse, though there was no unnatural adhesion. Interlobular emphysema existed throughout the lungs, which was manifested by bubbles of air collected on their surface, in clusters, and in strings or chaplets following the division of its lobuli. One of these strings traversed completely the circumference next to the ribs, of the left inferior lobe. There was also a considerable emphysema around the root of both lungs. Throughout the inferior lobe of each lung there was a high sanguineous congestion, such as exists in the acute stage of peripneumony, and which gave a solidity approaching to the sanguineous hepatization. This congestion was of a greater intensity at and about the root of the lungs. The remaining lobes were of a light spongy texture, and, except for their emphysematous state, seemed sufficiently fit to carry on respiration.

"The emphysema had passed into the anterior and superior mediastinum behind the sternum, and thence below the fascia profunda cervicis, into the root of the neck, up the trachea to the larynx.

"Having taken out all the respiratory organs together, and laid open the trachea and bronchia, there was found a perfect and entire lining of coagulating lymph, extending from the superior margin of the glottis, through the larynx, trachea, and bronchia, into the lungs. This membrane became thicker and thicker in its progress downwards; and could be traced satisfactorily into the secondary branches of the bronchia. It adhered with tenacity to the larynx, and upper half of the trachea; but not so much so as to prevent its being pulled off in a state perfectly distinct. In the lower part of the trachea, and in the bronchia, the membrane was so loose that it separated with the greatest facility, forming perfect tubuli.

"The mucous membrane of the larynx, trachea, and bronchia, beneath this lining, was highly injected with blood and inflamed, presenting an appearance, rather rougher than common. In the bronchia it was of a scarlet colour, which increased in intensity the further the bronchia penetrated into the lungs, until the ramifications became so small, as to prevent their being satisfactorily traced. The augmentation of colour was very abrupt, beyond the terminations of the lining membrane of lymph. The ramifications of the bronchia contained a sero-purulent fluid mixed with air."

From the absence of croupy symptoms till the 7th day of the disease which began as a simple catarrh, and from the false membrane being firm, thick, perfectly tubular, and nearly detached in the bronchia: whilst it was neither so thick, so readily detached, nor so uniformly diffused in the upper part of the trachea and larynx, Dr. Jackson believes that the inflammation and exudation commenced below and spread upwards. The emphysematous condition of the lungs so plainly indicated by the auscultic symptoms, was due, no doubt, to the obstruction offered to respiration by the croupy exudations. Bronchotomy would have done nothing, save mischief, in such a case.

*Case* 2. The younger sister of the preceding patient, aged 26 months, of good constitution, became feverish on the 8th of February, the day previous to the other's death. On examination the tonsils were found to be inflamed with slight ulcers on them, the respiration was quick and wheezing. It was put into the warm bath, and two grains of calomel exhibited every hour, but on the 9th the respiration was more difficult and croupy, the child more feverish. Thirty leeches to the throat, sinapisms to the legs, and the continuance of the calomel, with castor oil at 4 P. M. produced some relief to the symptoms. During the night, however, an aggravation took place, and next morning the croupy respiration was more severe, and the fever increased. Examination having now shewn the sister's lungs to have been extensively affected, forty leeches were applied to this child's thorax, and $\frac{1}{8}$th of a grain of antimony in water exhibited every half hour. A free emetic effect ensued, the symptoms became much abated, and at 7 P. M. the respiration in both lungs was found by the stethoscope to be clear and natural. All treatment was omitted, sago water given for nutriment on the 11th, and on the 12th all fever had disappeared. The voice did not perfectly recover for ten days after the disappearance of the disease. The following remarks of Dr. Jackson, though carried in their spirit a little too far, are still a hard hit at our calomel-and-opiumists.

"This case presented the commencement of the same train of symptoms that had conducted the preceding case to a fatal termination. The warm bath, purging, and leeches to the throat, had brought no amelioration to the condition of the patient: the symptoms of croup were unabated. They had failed, in conjunction with emetics and blisters, and the liberal exhibition of calomel, to rescue the sister from the grave. Instructed by the autopsy that had just been made of the body of the sister, the treatment was directed to the prompt reduction of inflammation of the bronchial mucous membrane, and of the lungs. A large number of leeches were applied to the thorax, and to the fossa above the sternum. An immediate amelioration ensued, and the ground, thus won, was maintained by powerful revulsion kept up on the gastric mucous tissue, by the free employment of the tartarized antimony.

"In this case we have another instance, in addition to the thousands furnished already by the practice of this country, that the remedies and the treatment of common inflammation are the best adapted to the cure of croup. What utility is there, then, in the designation of diptheritic, as employed by Bretonneau: of what service is the assumption of specific inflammations, in the pathology of diseases, when the means for the removal of common inflammation, are the most successful and the most appropriate for their treatment? It does not furnish a single fact available in the management of the disease; it does not present any positive idea as to the condition of organ or structure in which consists the disease; or determine a single principle as a guide to the practitioner. It is the persistence in this system, that continues to foster empiricism; to encourage the idle research, so long fruitlessly pursued, of specific remedies, and which are now preferred in croup; and throws our science into the vague and uncertain condition with which it has long been reproached."

Such sentiments, which we believe to be in a great measure just, would never do with the salivation party in this metropolis. With them, man, woman, or child are never safe whilst their teeth are in their heads, and be it high fever or low cachexia—acute rheumatism or slow dropsy—a chronic disturbance of the liver or a violent croup—Allah il A'lah, they cry, there is but one medicine, and that is mercury!

*Case* 3. M. R. a fine lad, six years of age, had enjoyed generally good health, with the exception of a severe attack of pleurisy and gastric inflammation when four years old, from which he recovered with difficulty. On the 5th of June, 1827, after malaise for some days, he was seized with fever attended by head-ache. He took some magnes. usta and magnes. sulph. at bed time, which opened his bowels. The fever continued on the 6th and he complained of sore throat; bled in the evening to 8 ozs. On the 7th the fever was less, but he had cough, expectorated fetid, puriform matter, and had epistaxis. On the 8th he became suddenly hoarse, and coughed up viscid phlegm of nearly membranous consistency—respiration easy—fauces red—tonsils enlarged, with slight ulceration. Took eight grs. of cal.—poultice to neck—warm bath. The bowels were freely opened, a profuse perspiration ensued, and the fever was much lessened in the evening. Next day, however, all the symptoms were again increased—croupy sound on coughing—ulcers on tonsils extended—posterior fauces covered with a white exudation. Fifty leeches to throat; ʒss. of solut. of ant. tart. every two hours—solution of argent. nit. (gr. xij. to ℥j. of water) applied by pencil to fauces; pediluvia.

10th. Croupy respiration—tonsils covered with exudation—no fever. Sinapism followed by blister to throat; cal. gr. ij. every hour; solution of caustic doubly strong applied to fauces; ant. tart. continued. He vomited frequently and was relieved throughout the day, but was awoke with strangling several times during the night. On the 11th the breathing was only stridulous at times. Ipec. gr. ¼ added to each calomel powder; fifty leeches to throat. The leeching was followed by improvement, and the exudation disappeared from the fauces—he vomited repeatedly during the day, and discharged a viscid mucus mixed with firm lymph. On the 12th, the improvement continued, and he took cal. gr. j. ipec. gr. ¼ every hour, with a dose of castor oil at 2 p. m. In the evening a febrile exacerbation took place, the respiration was more difficult and wheezing. Sixty leeches round the throat with hive syrup brought relief; he vomited frequently tenacious mucus. During the night he was restless, and on the 13th the respiration was laboured and harsh, the cough constant and severe, the sound that of a loose body in the trachea. Inhalation of vapour of vinegar, dose of calomel raised again to gr. ij. in the hour. From 11 o'clock to 3 the cough was incessant, and the little patient so conscious of some body rising and falling in the windpipe, that he repeatedly attempted to seize it with his fingers. Vomiting was procured by tartarized antimony, and some pieces of membrane discharged with immediate relief. He fell into a sleep from which he was awoke at 8 p. m. by cough;—respiration harsh—cough and strangling renewed—more membrane apparently loosened in the trachea, which sneezing from snuff would not dislodge. Vomiting was produced and a little tenacious mucus dislodged with the effect, as before, of procuring relief; the bowels were opened twice during the day. The following passage shews so well the terrible sequel of this interesting case, that we cannot refrain from quoting it entire.

" *14th.*—Terrible night; constantly threatened with suffocation, the danger of which became more and more pressing; the glottis appeared to be nearly closed by some mechanical obstruction; auscultation manifested healthy respiratory murmur in both lungs, showing all the difficulty to exist in trachea and larynx. This induced me to propose and urge bronchotomy as affording a chance of safety. At 1, Dr. J. R. Barton performed the operation of bronchotomy. The patient was then at the last gasp, and had ceased to respire about half a minute before it was completed. The operation was performed with Dr. Barton's usual skill, though the patient was convulsed from suffocation during its performance. On the trachea being opened, a gush of fluid took place from the wound, mixed with blood of a venous hue, and carrying with it some detached pieces of membrane; respiration was renewed through the aperture, and the blood discharged became florid. A mass of loosened membrane was seen in the trachea, part of which was removed by forceps introduced into the trachea, and the remainder was driven into the opening by a violent spasmodic effort of the muscles of respiration, in an attempt apparently to cough. It was immediately seized with the forceps and withdrawn. When unfolded, it exhibited the form of the trachea, its bifurcation, and several ramifications of the bronchia; relief was immediate; respiration natural in frequency and force; some wine and water were administered; in half an hour the colour of the lips became roseate; the animal heat was increased to its natural degree, and muscular strength renovated; respiratory murmur distinct and natural in both lungs; was cheerful, attempted to converse, sat up in bed, and favourable anticipations were indulged. The afternoon was passed in a comfortable state, with occasional sleep; at 8 p. m. pulse was more irritated; skin of febrile heat; respiration laboured; a muco-purulent discharge commenced from the aperture in the trachea; these symptoms continued to advance, the discharge became more viscid, and so abundant, it was difficult to keep the opening free; the respiration was more and more laborious; mucous ronchi were heard in every part of the chest; the sense of distress and suffocation augmented; relieved by coma, and finally death, at 5 a. m. of the 15th, seventeen hours after the operation."

*Dissection thirteen hours after death.*—Body thin, limbs slightly rigid.

"*Abdomen.* The stomach was distended; contained a quantity of clear fluid, principally the drinks he had taken previous to death; a thick, white, tenacious mucus, in considerable quantity, adhered to the lining or mucous membrane; in cardiac extremity this membrane was of brown colour; of whitish colour in other portions; thickness and consistency of the membrane no ways altered.

"The intestinal tube appeared in natural condition throughout, as respected colour, consistency, and thickness; secretions natural. Spleen and liver in normal state.

"*Thorax.* Right lung. The pleural surfaces adhered firmly in every part; this condition had resulted from the former severe attack of gastro-pleuritis. The bronchial tubes were filled with a serous frothy fluid which discharged from them when divided. Left lung. No pleural adhesions; collapsed; was crepitating; bronchial tubes contained frothy mucus in small quantities.

"*Trachea and larynx.* The interior of the larynx was completely incrusted over, with membraniform exudation; it lined the internal face of the epiglottis; the external was free of it; the rima glottidis was nearly closed by it. The ventricles of Morgagni, the ligaments, &c. were no longer apparent from the thickness of the membranous layer, spread over the interior of the larynx. This layer adhered firmly to the mucous membrane, but its surface was irregular, appearing as though pieces had been detached.

"The membraniform exudation extended through the trachea, and penetrated the bronchia, below its ramification, but did not enter the smaller divisions. At the ramification, the calibre of the bronchial tube was so much diminished as merely to admit a small quill. In the trachea, the exudation was closely adherent to the mucous membrane; in the bronchia it was detached and tapered off to a point. It could be separated, in the thickest portions, into layers, appearing to have been formed by successive exudations.

"The lower portion of the trachea and bronchia contained a fluid of the consistency and possessing the colour of cream; the tonsils and posterior fauces were covered with a copious, thick, viscid mucus, strongly resembling albumen. The fluid of the trachea, I suspect came from this source, and the alteration of its colour, proceeded from its mixture with the air. I observed before death, that every act of inspiration produced a singular rattling sound. The copious secretion from the fauces, could not be expectorated, in consequence of the opening into the trachea, which destroyed the phenomenon of coughing, and it was drawn into the trachea by the effort of inspiration. Hence the windpipe was filled with it, respiration embarrassed, and suffocation ensued from its accumulation in the trachea and bronchia, with that of the bronchial secretions, in the lungs, from the impossibility of expectorating them by the loss of the power of coughing."

The preceding cases appear to our author to justify the following conclusions:—

"1st. That inflammation of the tonsils is a frequent precursor to croup in children; and that it ought consequently always, in them, to be attentively watched, and should be treated very actively by the means best adapted to reduce inflammation.

"2d. That the membranous exudation may commence in the lower part of the trachea and the bronchia, extending upwards, and does not invariably arise in the fauces or larynx, and proceed downwards.

"3d. That the most prompt and decisive remedy for sanguine inflammation—that is, sanguineous depletion—is the only certain remedy, and should be resorted to in the first periods of the disease, so as to precede the membranous exudation.

"4th. That the membranous exudation having been once produced to any extent, little expectation of a recovery is to be entertained. When this result does occur, it is to be regarded as fortuitous, depending on some extraordinary circumstance, and which cannot be calculated as a probable event.

"5th. That when the membranous exudation has been thrown out in the larynx, trachea, and bronchia, the application of muriatic acid to the fauces, so highly extolled by Bretonneau, and of nitrate of silver, recommended by Dr. Mackenzie, from the local and limited impression they must necessarily make, can promise no beneficial operation—their influence cannot be extended to the surfaces from which the exudation takes place. This practice can be of utility only in rare cases, where the difficulty arises from an obstruction limited to the glottis and fauces.

"6th. That laryngotomy, or bronchotomy, is a useless operation, when the membranous exudation has actually occurred, and extends, as it mostly does, to the trachea and bronchia; or, when the inflammatory irritation of the respiratory mucous membrane is not subdued, and a free secretion from it is produced. The impossibility of coughing after the operation, prevents expectoration, the fluids accumulate in the trachea, bronchia, and bronchial ramifications in the lung, causing, finally, suffocation. The only case in which the operation promises success, is an obstruction confined entirely to the glottis, without disease prevailing to any extent in the respiratory mucous membrane."

The practical nature of the foregoing cases and their intrinsic value will form a sufficient apology for the lengthened notice we have given them. We believe that such articles as these are worth a thousand of the common flim-flams, termed original, of the day; in which it is the author's object to shew that he has made some wonderful discovery, the power of curing tetanus, for instance, by epsom salts, or the like. If such be physic, then, like Macbeth—We'll have none of it!

---

